# How Plants Sense Wounds: Damaged-Self Recognition Is Based on Plant-Derived Elicitors and Induces Octadecanoid Signaling

**DOI:** 10.1371/journal.pone.0030537

**Published:** 2012-02-09

**Authors:** Martin Heil, Enrique Ibarra-Laclette, Rosa M. Adame-Álvarez, Octavio Martínez, Enrique Ramirez-Chávez, Jorge Molina-Torres, Luis Herrera-Estrella

**Affiliations:** 1 Departamento de Ingeniería Genética, Centro de Investigación y de Estudios Avanzados (CINVESTAV), Irapuato, Guanajuato, México; 2 Laboratorio Nacional de Genómica para la Biodiversidad, Centro de Investigación y de Estudios Avanzados (CINVESTAV), Irapuato, Guanajuato, México; 3 Departamento de Biotecnología y Bioquímica, Centro de Investigación y de Estudios Avanzados (CINVESTAV), Irapuato, Guanajuato, México; National Taiwan University, Taiwan

## Abstract

**Background:**

Animal-derived elicitors can be used by plants to detect herbivory but they function only in specific insect–plant interactions. How can plants generally perceive damage caused by herbivores? Damaged-self recognition occurs when plants perceive molecular signals of damage: degraded plant molecules or molecules localized outside their original compartment.

**Methodology/Principal Findings:**

Flame wounding or applying leaf extract or solutions of sucrose or ATP to slightly wounded lima bean (*Phaseolus lunatus*) leaves induced the secretion of extrafloral nectar, an indirect defense mechanism. Chemically related molecules that would not be released in high concentrations from damaged plant cells (glucose, fructose, salt, and sorbitol) did not elicit a detectable response, excluding osmotic shock as an alternative explanation. Treatments inducing extrafloral nectar secretion also enhanced endogenous concentrations of the defense hormone jasmonic acid (JA). Endogenous JA was also induced by mechanically damaging leaves of lima bean, Arabidopsis, maize, strawberry, sesame and tomato. In lima bean, tomato and sesame, the application of leaf extract further increased endogenous JA content, indicating that damaged-self recognition is taxonomically widely distributed. Transcriptomic patterns obtained with untargeted 454 pyrosequencing of lima bean in response to flame wounding or the application of leaf extract or JA were highly similar to each other, but differed from the response to mere mechanical damage. We conclude that the amount or concentration of damaged-self signals can quantitatively determine the intensity of the wound response and that the full damaged-self response requires the disruption of many cells.

**Conclusions/Significance:**

Numerous compounds function as JA-inducing elicitors in different plant species. Most of them are, contain, or release, plant-derived molecular motifs. Damaged-self recognition represents a taxonomically widespread mechanism that contributes to the perception of herbivore feeding by plants. This strategy is independent of insect-derived elicitors and, therefore, allows plants to maintain evolutionary control over their interaction with herbivores.

## Introduction

How can plants recognize damage that is inflicted by herbivores? The discovery of the ‘wound response’ of tomato [1] initiated a series of studies to determine how local damage is perceived and translated into mobile signals that regulate a systemic resistance response [2], [3], [4]. Fragments of plant-derived molecules were among the first described defense elicitors and include peptides such as systemin [5] and cell wall-derived pectins, oligogalacturonides and oligosaccharides [6], [7], [8]. The early research therefore indicated that the plant itself contains the molecules that are required for defense induction. Concurrent research by several different research groups focused on the feeding insects and discovered specific herbivore-associated molecular patterns (HAMPs) [4], [9], [10], [11], including, for example, volicitin and other fatty acid–amino acid conjugates from the regurgitate of feeding caterpillars, bruchins and caeliferins [12], [13], [14], and plant-derived protein fragments that are formed specifically during insect feeding [15], [16], [17]. HAMPs boost resistance when applied to wounded plant tissues and usually elicit responses that differ from the response to mechanical damage.

However, assuming that plants use exclusively HAMPs to perceive that they are damaged by herbivores causes several conceptual problems. First, and most intriguingly, HAMPs are active only in certain insect–plant interactions [11], [18]. How do plants respond to the feeding damage caused by the majority of herbivores, including mammals? Second, it is unclear whether insect regurgitate is applied to plant tissue during the normal feeding process [19]. Finally, if a resistance induction is based only on the specific recognition of certain HAMPs, the herbivores, in principle, could change the molecular structure of their specific HAMPs and thereby avoid perception, or simply avoid applying their HAMPs to the wounded plant tissue [19]. By contrast, ‘damaged-self recognition’ would allow the plants to mount a general response to herbivory and retain evolutionary control over this vital process [20]. Recognition of the damaged self could be based principally on intact plant molecules localized outside their usual cell compartment or on fragments of plant molecules [20].

We investigated whether lima bean (*Phaseolus lunatus* L.) responds to chemical motifs that are indicative of the damaged self and whether the recognition of these signals triggers jasmonate signaling. Jasmonic acid (JA) plays a central role in activating the systemic defense after herbivory and in other vital processes, including flower development and senescence [3], and in floral nectar secretion [21]. One JA-dependent defense response is the secretion of extrafloral nectar (EFN), which attracts ants and other predatory insects as a means of indirect defense [22], [23]. We quantified EFN secretion in response to treatments that were likely to elicit damaged-self recognition and in response to chemically similar compounds that are not released from disintegrated plant cells. We also monitored endogenous JA synthesis and used pyrosequencing to compare the transcriptomic patterns induced in response to damaged-self signals with the response to JA. Damaged-self signals induced the synthesis of JA and the overall transcriptomic patterns induced by damaged-self signals in lima bean were similar to those induced by the hormone itself.

## Results and Discussion

### Induction of an indirect defense

**Figure 1:**
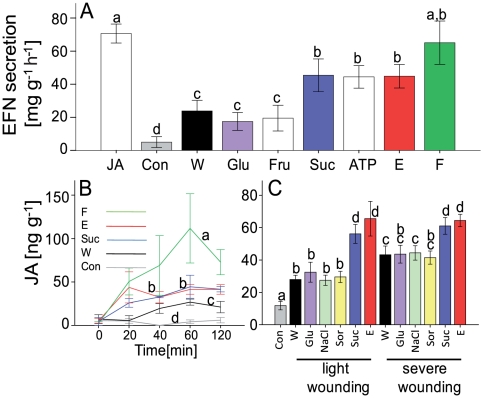
Damaged-self recognition in lima bean. (**A**) EFN secretion [mg soluble solids per gram leaf fresh mass and hour], (**B**) time course in endogenous jasmonic acid (JA) levels [ng per gram leaf fresh mass], (**C**) endogenous JA level 60 min after different treatments. Identical colors in different panels indicate identical treatments, values shown are means ± SE (of n = 7 biological replicates in panel A and n = 5 in panels B and C). Bars or lines marked with different letters indicate treatments that were significantly different (*P*<0.05) according to posthoc analysis with least significant difference tests. Abbreviations: Con = control, E = leaf extract, EFN = extrafloral nectar, F = flame wounding, Fru = fructose, Glu = glucose, Sor = sorbitol, Suc = sucrose, W = mechanical wounding.

Wild-type plants of *P. lunatus* were subjected to flame wounding, or to mechanical wounding with subsequent application of either water or 1 mM aqueous solutions of glucose, fructose, sucrose, ATP, or JA (positive control). EFN secretion quantified 2 h after treatment differed significantly among treatments (univariate ANOVA: F = 8.340, *P*<0.001, n = 7). Wounding alone increased EFN secretion over control levels but the response was significantly stronger after application of ATP, sucrose, leaf extract, or flame wounding. Glucose and fructose caused no further increase as compared to wounding alone (Fig. 1A). Sucrose is the major photosynthetic product and the dominant sugar in the phloem [24] and its extracellular concentration will inevitably increase upon tissue disruption, whereas neither glucose nor fructose is an abundant sugar in the leaf tissue. A sudden increase in the extracellular concentration of sucrose, but less so of the monosaccharides, would therefore appear to be a suitable indicator of severe tissue disruption. ATP is released from cells by secretion or by wounding and it has been suggested that ATP plays a role in plant defense signaling [25]. Flame wounding physically destroys entire cells, releasing their contents into the extracellular space. Flame wounding as applied in our study can induce proteinase inhibitor genes, which are classical markers of wound-induced genes whose expression depends on JA signaling [26], [27], and has been related to Ca^2+^ signaling [28], which is a well-known early step in the perception of herbivore-feeding by plants [29], [30]. Finally, leaf extract contains all the molecules that are released when cells become disrupted. Patterns in EFN secretion thus support the prediction that common plant molecules or their fragments are monitored in the extracellular space for damaged-self recognition in lima bean.

### Induction of endogenous JA

**Figure 2:**
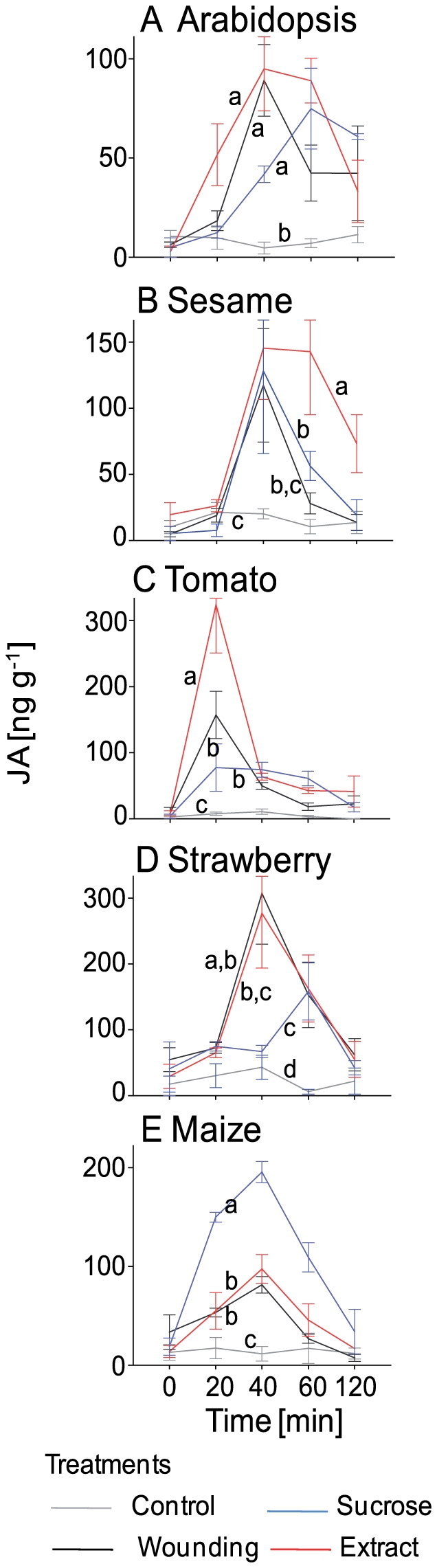
Endogenous levels of jasmonic acid (JA). Endogenous JA levels were quantified (ng per gram leaf fresh mass) at different times after the treatment in (**A**) Arabidopsis, (**B**) sesame, (**C**) tomato, (**D**) strawberry, and (**E**) maize. Lines marked with different letters indicate statistically significant differences among treatments (least significant difference post hoc tests, n = 5 biological replicates per mean).

We applied the above treatments and quantified endogenous JA using gas chromatography coupled with single-ion mass spectrometry (GC-SIM-MS). Wounding induced endogenous JA, but leaf extract, sucrose and flame wounding had significantly greater effects (Fig. 1B, Table 1). The effect of wounding alone was dependent on the severity of the damage, and glucose, sorbitol or salt (NaCl) at 1 mM had no significant effect on the induction of endogenous JA compared with the levels of JA induced by wounding alone (Fig. 1C), which excludes osmotic shock as the reason for the JA response. Similar effects were observed in other plant species. Mechanical wounding of leaves of *Arabidopsis thaliana* (Brassicaceae), tomato (*Solanum lycopersicum*, Solanaceae), strawberry (*Fragaria×ananassa*, Rosaceae), sesame (*Sesamum indicum*, Pedaliaceae) and maize (*Zea mays*, Poaceae) caused a significant induction of endogenous JA in all species compared with the level of endogenous JA in intact leaves (Fig. 2, Table 1). The application of sucrose induced the greatest increase in endogenous JA levels in maize, whereas in sesame and tomato the application of leaf extract induced a greater increase in JA concentration than that induced by mechanical damage (Fig. 2), confirming earlier reports of an induction of JA-dependent plant defenses after the application of leaf extract [1], [31], [32], [33]. Therefore, both the amount of damage (Fig. 1C) and the species (Fig. 2) can affect whether any of the damaged-self signals boost the JA response above the levels that are seen after mere mechanical damage. We conclude that all the plants investigated here can respond to mechanical damage to some degree, whereas the specific signals perceived and the degree of the response varies among species. Damaged-self recognition represents a taxonomically common mechanism that is subject to physiological and evolutionary flexibility.

**Table 1:** Results of ANOVA for levels of endogenous JA.

Species	Factor	df	*F* value	*P* value
**Lima bean**	Treatment	4	13.730	<0.001
	Time	4	7.494	<0.001
	Treatment×time	16	1.632	0.096
**Sesame**	Treatment	3	7.289	<0.001
	Time	4	11.087	<0.001
	Treatment×time	12	1.745	0.073
**Tomato**	Treatment	4	15.188	<0.001
	Time	3	25.347	<0.001
	Treatment×time	12	7.296	<0.001
**Arabidopsis**	Treatment	4	21.302	<0.001
	Time	3	18.077	<0.001
	Treatment×time	12	5.777	<0.001
**Strawberry**	Treatment	4	8.661	<0.001
	Time	3	10.652	<0.001
	Treatment×time	12	2.822	0.003
**Maize**	Treatment	3	51.031	<0.001
	Time	4	31.361	<0.001
	Treatment×time	12	8.418	<0.001

ANOVA was conducted separately for every species on the effects of treatment and time as fixed factors on endogenous levels of JA.

### Transcriptional changes after damaged-self recognition

The application of leaf extract or sucrose induced a greater increase in endogenous JA than that induced by mere wounding in lima bean, sesame, tomato and maize, and mere wounding increased endogenous JA over control levels in all species investigated here (Figs. 1 and 2). In lima bean, an increase in the severity of the wounding led to an increase in the level of endogenous JA that was dependent on the severity of the damage, whereas the application of leaf extract or sucrose elicited a strong JA response that was independent of the damage level applied before (Fig. 1C). Finally, several different damaged-self signals were able to induce EFN secretion by lima bean (Fig. 1A). All these observations appear contradictory to reports that HAMPs are required for a full resistance response [5], [9], [10], [12], [14], [15], [16], [18], [33], [34], [35]. To resolve this apparent contradiction, we must consider the quantitative aspect: studies aimed at elucidating the particular role of damaged-self recognition in the overall response to herbivore feeding could compare endogenous JA concentrations after different damage levels (with and without the application of leaf extract) to the levels observed after damage caused by different types of herbivores, and they should consider the effects downstream of JA. Our preliminary results indicate that the relative importance of damaged-self recognition is likely to vary among species.

How similar are transcription patterns after damaged-self recognition to the full set of JA-dependent transcriptional responses in lima bean leaves? We used 454 pyrosequencing [36] of cDNA libraries to investigate transcriptomic changes (in relation to the transcriptome of untreated control plants) after JA treatment, flame wounding, mechanical wounding, and mechanical wounding with subsequent application of leaf extract or sucrose. We extracted mRNA, produced cDNA, tagged the resulting libraries and then subjected them to two sequencing runs [37]. During 454 sequencing, the number of sequencing reads representing the same gene can be taken as a measure of transcript abundance (see Text S1).

**Figure 3:**
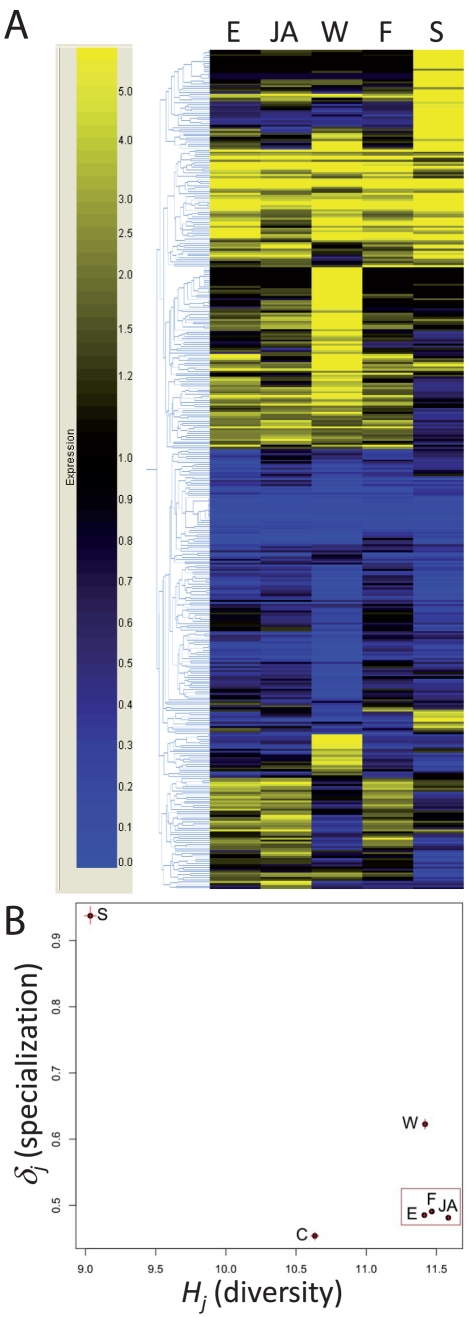
Transcriptomic response of lima bean leaves to damaged-self signals and jasmonic acid (JA). (**A**) Transcriptomic patterns (genes up- and downregulated in comparison to untreated controls) are presented for JA treatment (JA) in comparison to flame wounding (F), mechanical wounding (W), and mechanical wounding with subsequent application of leaf extract (E) or 1 mM solution of sucrose (S). (**B**) Scatter plot of *Hj* (diversity) versus δ*j* (specialization) for each treatment tested.

The overall patterns of up- and down-regulated genes (see Table S1 for the list of contigs, accession numbers, annotation results and expression levels of differentially expressed genes) as compared to the control were surprisingly similar among lima bean leaves that had been treated with JA and leaves that had been subjected to fire or to mechanical wounding with subsequent application of leaf extract (Fig. 3). By contrast, mechanical wounding alone and mechanical wounding with subsequent application of sucrose solution caused clearly distinct patterns (Fig. 3).

Leaf extract application and particularly flame wounding elicited very similar transcriptomic patterns to those elicited by JA (Fig. 3A). Forty-three genes were induced and 71 were repressed by all three treatments. The transcriptome elicited by JA overlapped with a further 48 genes (29 up, 19 down) in the wounding-induced transcriptome and with 36 genes (33 up, 3 down) in the extract-induced transcriptome (Table 2). According to a Gene Ontology classification using the BioMaps tool from the VirtualPlant webpage (http://virtualplant.bio.nyu.edu/cgi-bin/vpweb/) [38] most of the upregulated genes represent categories such as defense and virulence, interaction with the environment and responses to wounding or stress (Table S2). Several of the highly upregulated genes (by factors >10) are involved in JA synthesis (e.g. *LOX*, *lipoxygenase*; *AOS*, *allene oxide synthase*; and *AOC*, *allene oxide cyclase*) [3] or in the regulation of JA-isoleucine-responsive genes downstream of JA (*JAZ1*, *jasmonate ZIM-domain*) [39], [40], [41]. Genes related to photosynthesis or primary metabolism appeared downregulated, which is in line with the repressing effect of JA on photosynthesis and other growth-related processes [42]. Only a few of the induced genes were related to osmotic or salt stress (Table S3), which further supports our view that our treatments caused a specific response rather than general osmotic stress.

**Table 2:** Differentially expressed genes in response to wounding, JA and leaf extract.

	Genes	Significant response to		
		E	JA	W
Upregulated	43	+	+	+
	33	+	+	0
	29	+	0	+
	14	0	+	+
	63	0	0	+
	7	+	0	0
	26	0	+	0
Downregulated	71	−	−	−
	3	−	−	0
	19	−	0	−
	13	0	−	−
	33	0	0	−
	5	−	0	0
	5	0	−	0

Numbers of genes are indicated that responded with a significant up (+) or down (−) regulation to at least one of the treatments. Genes were regarded as differentially expressed when their expression was ≥2.0 or ≤0.5 as compared to the untreated control. E = extract, JA = jasmonic acid, W = mechanical wounding.

**Figure 4:**
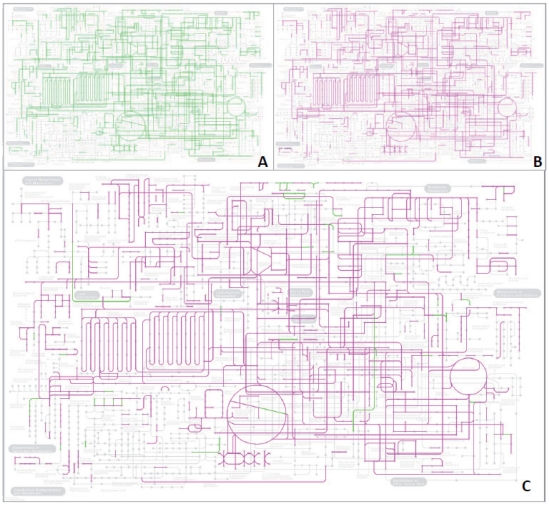
Metabolic pathways represented in the *Phaseolus lunatus* unigene set. Global metabolism map constructed combining existing pathway maps and corresponding genes referenced in the KEGG database for *Arabidopsis*, *Populus*, *Vitis*, *Oryza* and *Sorghum* (green lines). (**B**) Global metabolism map represented by the *P. lunatus* unigene set (magenta lines). (**C**) Overlap comparison of the KEGG metabolic global map of flowering plants (*Arabidopsis*, *Populus*, *Vitis*, *Oryza* and *Sorghum*) with the metabolic map represented by the *P. lunatus* unigene set.

Because the metabolic pathways that we obtained from the *P. lunatus* unigene set yielded an almost complete coverage of a global metabolic map according to the Kyoto Encyclopedia of Genes and Genomes (KEGG) classification (Fig. 4), we are confident that our transcriptome is sufficiently complete as to allow conclusions concerning global changes in gene expression. To quantify the similarities among these transcriptomes, we compared their diversity (*H_j_*) and specialization (*δ_j_*), defining *H_j_* as the Shannon entropy of the frequency distribution of a transcriptome and *δ_j_* as the average specificity of the genes expressed under each condition [43]. Plotting the transcriptomes in a two-dimensional space defined by *H_j_* and *δ_j_* as well as calculating Euclidean distances among the distributions of the transcriptomes confirmed that the transcriptomes obtained after JA treatment, leaf extract application and flame wounding clustered closely together (Fig. 3B, Table 3). Apparently, the global changes in gene expression after the application of leaf extract after mechanical wounding or after damaging the tissue via flame wounding were mediated via jasmonate signaling.

**Table 3:** Euclidean distances between lima bean leaf transcriptomes in a two-dimensional space defined by δj (specialization) and Hj (diversity).

	C	E	JA	W	F
**E**	0.0235				
**JA**	0.0342	0.0197			
**W**	0.0524	0.0404	0.0284		
**F**	0.0224	0.0151	0.0223	0.0452	
**S**	0.0954	0.0958	0.1047	0.1113	0.10127

C: control, E: leaf extract, JA: jasmonic acid, W: mechanical wounding, F: flame wounding, S: sucrose.

Mere mechanical wounding overall elicited similar transcriptomic patterns but was clearly distinguishable from the transcriptomic patterns elicited by JA, flame wounding, or leaf extract application after mechanical wounding. We observed a set of genes which were either induced only after wounding or that were repressed by wounding but induced by JA, or when wounding was caused by fire or complemented by the application of leaf extract (see lower parts in Fig. 3A). Several of the genes that were repressed by mechanical wounding alone, but not repressed by JA or flame wounding or when leaf extract was applied to the wounded leaves, appeared to be related to photosynthesis (Table S1). Genes that were induced at least fourfold by mechanical wounding, but not induced by leaf extract application to wounded leaves, or by JA application or flame wounding, appeared, among others, to have ATPase and other ATP binding functions (Table S1).

Mechanical damage as applied in our study is likely to release some damaged-self signals. However, the transcriptomic patterns elicited by our mechanical wounding treatment differed in some parts from the patterns that resulted when the entire contents of many cells were applied to the wounded tissue. Sucrose caused gene expression alterations that overlapped partly, but in general differed from the gene expression induced by the other treatments (Fig. 3). Although sucrose induced both EFN secretion and endogenous JA-levels in lima bean (Fig. 1), sucrose-signaling is also involved in multiple independent physiological responses [24], [44]. It therefore appears to be likely that an exogenous application of sucrose induced a mixed response whose components can be related to both damaged-self recognition and other physiological processes.

**Figure 5:**
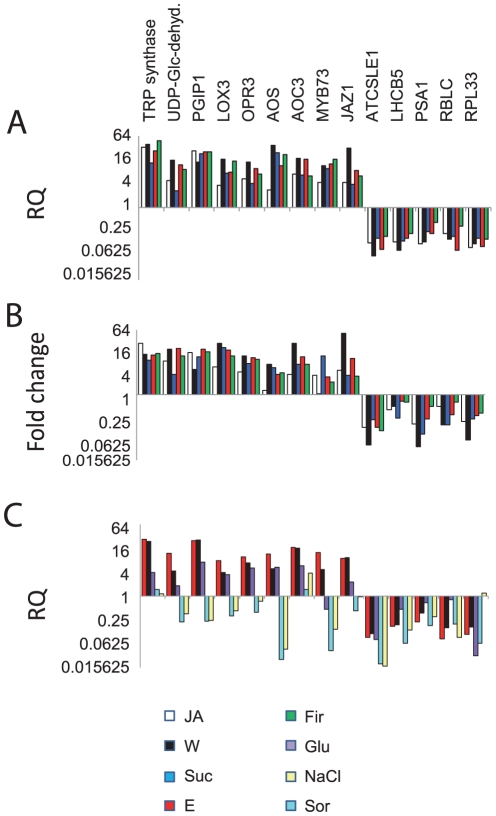
Expression of functionally important genes. Expression patterns of *tryptophan synthase*, *UDP-glucose-6 dehydrogenase*, *PGIP1* (*polygalacturonase-inhibiting protein 1*), *LOX3* (*Lipoxygenase3*), *OPR3* (*12-oxophytodienoate-reductase 3*), *AOS* (*allene oxide synthase*), *AOC3* (*allene oxide cyclase 3*), *MYB73* (*MYB domain protein 73*, transcription factor), *JAZ1* (*jasmonate-ZIM-domain repressor protein 1*), *ATCSLE1* (*cellulose synthase/transferase*), *LHCB5* (*light harvesting complex of photosystem II subunit 5*), *PSA1* (*photosystem I subunit I*), *RBCL* (*large subunit of Rubisco*) and *RPL33* (*chloroplast ribosomal protein 33*) are presented, obtained with qRT-PCR (A) and 454 sequencing (B). Panel (C) depicts qRT-PCR results in response to control treatments (glucose, NaCl and sorbitol). Bars in panel (A) and (C) present means of three independent PCR runs. See Table S1 for unigene identities, accession numbers, annotation results and expression levels, and Table S4 for primer sequences. Treatments applied were: E = leaf extract, F = flame wounding, Glu = glucose, JA = jasmonic acid, NaCl = sodium chloride, Sor = sorbitol, Suc = sucrose, W = mechanical wounding.

**Figure 6:**
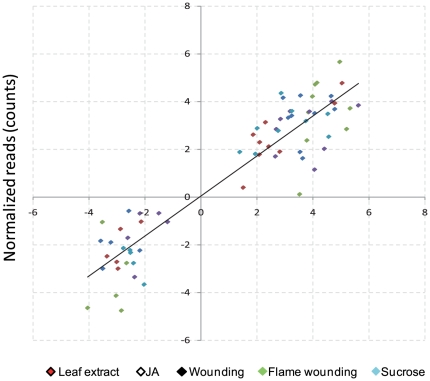
Correlation of expression levels as obtained from 454 sequencing with those obtained with qRT-PCR.

The expression patterns of 14 genes were confirmed with quantitative reverse-transcriptase polymerase chain reaction (qRT-PCR, see Table S4 for primer sequences). We found a good correlation between expression levels obtained by 454 sequencing and those obtained by qRT-PCR (Figs. 5 and 6). Interestingly, leaf extract, sucrose and flame wounding treatments induced the expression of *AOS*, *AOC*, *LOX*, *OPR* and *JAZ1* more strongly than JA itself (Fig. 5). At the phenotypic level, the same treatments induced high levels of endogenous JA in lima bean, with flame wounding eliciting the highest levels, a result that is fully congruent with the strong induction of EFN secretion that occurred following this treatment (Fig. 1). NaCl and sorbitol induced gene expression patterns that were the opposite of those induced by the damaged-self signals. The level of induction of most of the up-regulated genes was much lower following glucose application than for the other treatments, and MYB73, a transcription factor that was induced by all damaged-self signals, including sucrose, was repressed by glucose (Figure 5C). Although the signals that trigger the defense response represent common plant molecules (or changes in their extracellular concentration), the specificity that we observed in the transcriptomic changes appears to be high enough to allow a fine-tuning of the response.

### Conclusions

Plants require a general wound-recognition system that is independent of the detailed nature of the attacking herbivore and that cannot be controlled by the animal at the physiological or evolutionary level. Damaged-self recognition is based on the detection of plant-derived molecules and, thus, does fulfill these requirements.

Plants are likely to use both strategies, damaged-self recognition and HAMP-based specific responses, to optimize their response to herbivore feeding [4]. In our study, the application of leaf extract to slightly wounded lima bean leaves and flame wounding elicited almost the same gene transcriptional patterns as those elicited by JA. The similarities among gene expression patterns seen after the application of leaf extract and after flame wounding indicate that the systemic induction of proteinase-inhibitor genes after flame wounding [26], [27], [45], [46] is likely to be an effect of the cellular events that are common to both folivory and strong heat stress: the rapid release of the cellular content into the extracellular space and the disruption of plant macro-molecules. The transcriptomic patterns elicited by mechanical damage alone differed in some parts from the patterns that resulted when the entire contents of many cells were applied to the wounded tissue, indicating that damaged-self recognition allows specific responses without the need for insect-derived elicitors. Common molecules such as ATP and sucrose induced JA synthesis, whereas chemically similar molecules that are not released from damaged plant cells did not. When occurring at high concentrations in the extracellular space, sucrose, ATP and fragments of plant macromolecules can indicate the presence of disrupted cells.

Damaged-self recognition in plants shows surprising similarities to the role played by extracellular ATP in wound perception in the human skin [47]. Monitoring fragments of molecules, or molecules that are localized outside their normal compartment, could thus be a common principle by which organisms detect injuries. For plants, we suggest that damaged-self recognition, which we found in taxonomically unrelated species from both the monocots and the dicots, represents an evolutionarily ancient mechanism that allows a general response to herbivory without depending on animal-derived elicitors. Evolutionarily more derived mechanisms that monitor specific animal-derived molecules might then allow faster and more intensive responses to encounters with specialist enemies.

## Materials and Methods

### EFN secretion

Plants of *P. lunatus* growing at their natural site (México, Pacific coast, ∼15°55′N and 97°09′W) were assigned to groups of seven and subjected to the following treatments: control, flame wounding (placing the flame of a lighter at distance of 3 cm below the leaf tip for 2 sec, see refs. [26], [27], [45], [46]), mechanical damage (entire leaves were slightly wounded with a metal brush, causing approximately five punctures with a diameter of 0.1 mm per cm^2^, and then immediately sprayed with water) and mechanical damage followed immediately by the application of leaf extract (*ca.* 0.5 g fresh leaf material per ml water homogenized in a mortar, left to sediment and supernatant then used without further preparation) or of a 1 mM aqueous solution of glucose, fructose, sucrose, ATP, or JA. EFN secretion was quantified 2 h later using microcapillaries and a portable refractometer [23].

### Endogenous JA levels

Plants of all species were cultivated in a greenhouse for six weeks and then subjected to the following treatments: control, mechanical damage, and mechanical damage followed by the application of leaf extract or sucrose (lima bean was also subjected to the flame wounding treatment). In a second experiment, lima beans leaves were subjected to two different levels of damage (ca. 2 and ca. 8 punctures per cm^2^) followed by the application of leaf extract or a 1 mM aqueous solution of sucrose, glucose, sorbitol or NaCl. 500 mg of leaf material was collected immediately before treatment and at 20, 40, 60 and 120 min after treatment (second experiment with lima bean: 60 min only), stored in liquid nitrogen and extracted with ethyl acetate [48]. After adding [9,10-H_2_] dihydrojasmonic acid as an internal standard, JA was derivatized by adding 10 µl pentafluorobenzyl bromide and resuspended in 100 µl methanol [49]. One microliter of the sample was injected in the splitless mode and analyzed by gas chromatography-single ion-mass spectrometry in an Agilent Technologies Gas Chromatograph 7890A using a DB-1MS column (60 m×0.25 mm×0.5 µm Agilent Technologies) coupled to a MSD 5973 detector in SIM mode for 141, 181, 390 and 392 m/z. Other gas chromatography and mass spectrometry conditions were as described previously [50].

### Transcriptomic analyses

Leaves were treated (control, JA treatment, flame wounding, mechanical damage, and mechanical damage followed by application of leaf extract or sucrose) and collected 120 min later for mRNA extraction using Trizol® and Qiagen RNAeasy columns (Qiagen, México D.F.). Multiplex IDentifiers were used to tag cDNA libraries. Two FLX pyrosequencing runs were performed for all six conditions. A total of 955,244 reads were generated with an estimated average size of 217 bases representing a total of ≈207.52 Mbp. Sequences were assembled using Newbler (v1.1.03.24) software, producing a total of 44,653 singletons and 25,803 contigs. Every contig comprised on average 35 reads and had 396 bp. A *P. lunatus* unigene set was generated by combining 25,783 assembled contigs and 18,373 non-assembled reads (singlets), considering sequences with a minimum size of 100 bp. The unigene set was annotated by searching for sequence similarities using BLASTx against *Arabidopsis thaliana* (TAIR v9.0) gene models. The expression profiles from 454-sequencing were achieved by counting the number of sequencing reads per unigene, in this case represented by the number of BLASTn query 454 reads for each target unigene ID in each of the six samples (control, JA treatment, flame wounding, mechanical damage, and mechanical damage followed by application of leaf extract or sucrose). The frequency at which the sequence of a given gene is read in expressed sequencing tag projects should reflect the relative abundance of the corresponding mRNA [51], [52], [53]. See Text S1 for detailed methods.

### Ethics statement

Because lima bean is a wild species and all the authors work in Mexican Institutions, no permits were required to realize the field experiments.

## Supporting Information

Table S1 Differentially expressed genes in the *Phaseolus lunatus* transcriptome. Complete list of those 406 non-redundant unigenes (contigs, accession numbers, annotation results and expression levels) that exhibited putative differential expression in at least one condition evaluated.
(XLS)

Table S2 Over-represented functional categories in the *Phaseolus lunatus* transcriptome. The over-represented functional categories were defined according to the MIPS classification using BioMaps tool from the VirtualPlant webpage (URL: http://virtualplant.bio.nyu.edu/cgi-bin/vpweb/). Hypergeometric method and a Bonferroni correction were used for the analysis with a cutoff of 0.01.
(XLS)

Table S3 Expression profiles of genes represented in the “osmotic and salt stress response” MIPS categories.
(XLS)

Table S4 Primer sequences used for qRT-PCR.
(XLS)

Text S1 Annotation of the *Phaseolus lunatus* ESTs. A detailed explanation of all bioinformatic steps taken during the analyses of the transcriptomic data presented in this study.
(DOC)
