# Supplementary material for: How Plants Sense Wounds: Damaged-Self Recognition Is Based on Plant-Derived Elicitors and Induces Octadecanoid Signaling
Source: PLoS One. 2012 Feb 9;7(2):e30537. doi: 10.1371/journal.pone.0030537 (PMC3276496; doi:10.1371/journal.pone.0030537)
Supplement: Text S1 — Annotation of the Phaseolus lunatus ESTs. A detailed explanation of all bioinformatic steps taken during the analyses of the transcriptomic data presented in this study. (DOC) [file pone.0030537.s005.doc]

**Annotation of the *Phaseolus lunatus* ESTs**

A *P. lunatus* unigene set was generated by combining 25,783 assembled contigs and 18,373 non-assembled reads (singlets). The unigene set was annotated by searching for sequence similarities using BLASTx against proteins of both dicotyledonous and monocotyledonous plants [*Arabidopsis thaliana* (TAIR v9.0), *Populus trichocarpa* (JGI v1.1), *Ricinus communis* (TIGR v1.0), *Vitis vinifera* (PlantGDB v1.0), *Oryza sativa* (TIGR v6.1) and *Sorghum bicolor* (JGI v1.0)]. The likelihood of finding similarity to previously described proteins is highly dependent on the length of the query sequence. Comparisons of similar nature (1) have, however, demonstrated that query sequences of >100 bp length can ususally be annotated and reveal at least one highly significant BLASTx hit (E-value 10-5). In our data set, out of the 27,327 unigenes being longer than 100 bp, 16,910 (61.88%) had similarity to at least one

protein model at an E-value of 10-5. Next, we determined the proportion of annotated gene models for which homology was detected among *P. lunatus* unigenes measuring over 100 bp. Homology was detected to 51.81% of *Vitis* (12,171 of 23,493), 40.38% of *Oryza* (10,879 of 26,940), 37.40% of *Ricinus* (11,676 of 31,221), 37.23% of *Arabidopsis* (12,437 of 33,405), 33.62% of *Populus* (14,236 of 42,344) and 30.24% of *Shorgum* (10,585 of 35,005) gene models (E-value 10^-5^). Using the KEGG Atlas (<http://www.genome.jp/kegg/atlas/>) we created a global metabolism map combining 149 existing pathway maps and corresponding to 10,285 genes referenced to in the KEGG database for *Arabidopsis, Populus, Vitis, Ricinus, Oryza* and *Shorgum*. This metabolism map was compared to the global map created for the *P. lunatus* unigene set (147 distinct metabolic pathways assigned) (SI Figure 5). Overall, these results indicate that the *P. lunatus* unigene set yields a deep representation of the complex metabolic pathways that characterize a plant genome and can be used as a new source of genomic information to explore the structural, functional and evolutionary diversity of the *Fabaceae*.

**Expression analysis in *P. lunatus***

In principle, the frequency at which the sequence of a given gene is read in EST sequencing projects should reflect the relative abundance of the corresponding mRNA. Following this principle, the expression profiles in the transcriptomes that were obtained using 454-sequencing were achieved by counting the number of transcripts per gene, in this case represented by the number of BLASTn query 454 reads for each target Unigene ID in each of the six samples (C, W, E, S, F and JA). Trial and error tests, varying the size of the alignment and evaluating the identity percent of the aligned segments, were used to determine that the most common error found in the 454 reads was the deletion or insertion of, on average, one base every 30 nucleotides, which is consistent with previously reported observations (2). Thus, for our analysis we considered as a significant hit all reads showing an alignment ≥ to 30 nt with an identity ≥ 96.6%. We used Fisher’s exact test for independence in a 2 by 2 contingency table to compare the frequency of transcription of each gene in pairs of cDNA libraries as suggested previously (see, for example, ref. 3). We estimated that the results using a p-value ≤ 6.89E-6 could be considered significant (α=0.1; probability of Error Type I, using the Bonferroni correction for multitesting). Using this threshold, we identified 300 unigenes with putative differential expression when W and C were compared. The p-value threshold is dependent of the data set size. We, therefore, estimated the p-values that could be considered significant (α=0.1) in each library (E, JA, F and S) in a comparison with C (p-values: ≤ 4.43E-06; 4.26E-06; 3.37E-06 and 8.74E-06, respectively). We identified 406 non-redundant unigenes with putative differential expression in at least one condition evaluated (E, JA, W, F or S). For this sub-set of genes showing purportedly a differential expression, the relative abundance (RA) value for a given gene in a given sample was calculated as the 454-read counts for the gene in question divided by the total number of 454-read counts for the 406 genes in the sample, and the AR values where normalized in the R environment (R Development Core Team 2004, Version 2.1.1; http://www.r-project.org) using “Stable genes-Based Quantile” (SBQ) normalization (4). The SBQ-normalization compensates for the low number of genes to be normalized. To implement this method we used the expression profiles of 38 stably expressed genes. We constructed the stably expressed genes set choosing all unigenes that appeared being homologues (E-value < 10^-5^) to *Arabidopsis thaliana* housekeeping proteins (ACT 11, ACT 2/7, CYP2, TUA, TUB, UBQ11, UBQ7, EIF4B-L and EIF1β, see ref. 5). Finally, the fold change was estimated as the ratio between the relative abundance values (normalized) calculated for each gene in the conditions that were compared to C (E, JA, W, F and S). Ratio values of ≥ 2-fold and ≤ 0.5-fold were considered as indicating differentially expressed genes. In order to add further statistical support to our list of putatively differentially expressed genes, we re-analyzed our normalized data with the SAM (significance analysis of microarrays) package (6). Using 10% false discovery rate (FDR) as a cutoff, the results obtained by SAM confirmed the expression level of 92% of the unigenes that had been selected by Fisher’s exact test (data not shown).

**References**

1. Novaes E*, et al.* (2008) High-throughput gene and SNP discovery in *Eucalyptus grandis*, an uncharacterized genome *BMC Genomics* 9:312.

2. Huse SM, Huber JA, Morrison HG, Sogin ML, Welch DM (2007) Accuary and quality of massively parallel DNA pyrosequencing. *Genome Biology* 8:R143.

3. Kanji GK (1993) *100 Statistical Tests* (Sage Publications, London, UK) p 110.

4. Sato M*, et al.* (2007) A high-performance, small-scale microarray for expression profiling of many samples in Arabidopsis-pathogen studies. *Plant J* 49:565-577.

5. Brunner AM, Yakovlev IA, Strauss SH (2004) Validating internal controls for quantitative plant gene expression studies. *BMC Plant Biology* 4:14.

6. Tusher VG, Tibshirani R, Chu G (2001) Significance analysis of microarrays applied to the ionizing radiation response. *Proc Natl Acad Sci USA* 98:5116–5121.
